# Combined effect of pancreatic lipid content and gene variants (*TCF7L2*, *WFS1* and *11BHSD1*) on B-cell function in Middle Aged Women in a Post Hoc Analysis

**DOI:** 10.1186/s13098-022-00876-z

**Published:** 2022-07-27

**Authors:** Ákos Nádasdi, Viktor Gál, Tamás Masszi, Attila Patócs, Peter Igaz, Anikó Somogyi, Gábor Firneisz

**Affiliations:** 1 Department of Internal Medicine and Haematology, Faculty of Medicine, Semmelweis University, Szentkirályi St 46, 1088 Budapest, Hungary; 2 Brain Imaging Centre, Research Centre for Natural Sciences, Eötvös Loránd Research Network, Budapest, Hungary; 3 MTA-SE Hereditary Tumors Research Group, Eötvös Loránd Research Network, Budapest, Hungary; 4 Department of Laboratory Medicine, Faculty of Medicine, Semmelweis University, Budapest, Hungary; 5 MTA-SE Molecular Medicine Research Group, Eötvös Loránd Research Network, Budapest, Hungary; 6 Department of Endocrinology, Faculty of Medicine, Semmelweis University, Budapest, Hungary; 7 Department of Internal Medicine and Oncology, Faculty of Medicine, Semmelweis University, Budapest, Hungary

**Keywords:** Pancreatic fat, Liver fat, *PNPLA3*, *TCF7L2*, *WFS1*, *11ΒHSD1*, HbA1c

## Abstract

**Background:**

*TCF7L2* rs7903146 and *PNPLA3* rs738409 gene variants confer the strongest risk for type 2 diabetes mellitus (T2DM) and non-alcoholic fatty liver disease (NAFLD), respectively. Pancreatic triacylglycerol content (PTGC) was reported to have a role in T2DM development. We aimed to assess the correlation between PTGC and hepatic triacylglycerol content (HTGC) stratified by *PNPLA3* rs738409 genotype and subsequently interactions between PTGC and gene variants associated with β-cell dysfunction (*TCF7L2*, *WFS1*) and visceral adiposity (*11ΒHSD1*) on β-cell function were also tested.

**Methods:**

PTGC and HTGC were assessed using MR in a post-hoc analysis of a genotype-based (*PNPLA3* rs738409) recall study of 39 (lipid- and glucose lowering) drug-naïve women. Oral glucose tolerance test, HbA1c, insulin indices, anthropometric data were evaluated. The effect of minor allele carrying of *TCF7L2* (rs7903146); *WFS1* (rs1801214) and *11ΒHSD1* (rs4844880) variants in combination with PTGC was studied on surrogate markers of β-cell function. We used Spearman’s rank-order, Mann-Whitney-U tests, and linear regression models.

**Results:**

PTGC and HTGC values were correlated after stratification by the rs738409 variant (only in *CC* genotype group R = 0.67, p = 10^− 4^). PTGC and HbA1c values correlated in the entire study population (R = 0.58, p = 10^− 4^). Insulin resistance, sensitivity and disposition indices were correlated with PTGC (HOMA2-IR: R = 0.42, p = 0.008; TyG: R = 0.38, p = 0.018; Matsuda: R= − 0.48, p = 0.002; DI_basal_: R=−0.33, p = 0.039; ISSI-2: R=−0.35, p = 0.028). Surrogate markers of β-cell function (HOMA2-B, AUC_insulin_/AUC_glucose_) correlated significantly with PTGC in subjects with the following genotypes rs7903146: *CC* R = 0.51, p = 0.022; rs18001214: *CT* + *CC* R = 0.55, p = 0.013; rs4844880: *TA* + *AA* R = 0.56, p = 0.016. The strongest interactions were found between PTGC and *TCF7L2* rs7903146 effect on HOMA2-B (p = 0.001) and AUC_insulin_/AUC_glucose_ (p = 0.013).

**Conclusions:**

The *PNPLA3* rs738409 genotype has a major effect on the correlation between PTGC and HTGC. Furthermore we first report the combined effect of PTGC and individual risk gene variants of *TCF7L2*, *WFS1* and *11ΒHSD1* on β-cell dysfunction. The correlation between pancreatic lipid accumulation and HbA1c also indicates an important role for the latter pathology.

## 1. Introduction

Fatty pancreas was first reported by Ogilvie in 1933 in patients with obesity including a few individuals with type 2 diabetes mellitus (T2DM) [1].

Despite the global burden of obesity and related diseases including T2DM and non-alcoholic fatty liver disease (NAFLD) the role of fatty pancreas regained interest in the study of the development of metabolic diseases recently [2, 3]. There is an increasing number of supporting data that the accumulation of pancreatic fat has a role in the development of T2DM and related metabolic disorders [4]. Moderate evidence suggests that the reduction in pancreatic fat improves the metabolic condition, including the improvement of beta cell function and even potential reversal of T2DM after bariatric surgery [4, 5].

In contrast to NAFLD that is characterized with an internationally accepted cut-off value of 5.5% liver lipid content determined by magnetic resonance (MR) based methods [6, 7], currently there is a lack of both clinically meaningful standardized examination methods and internationally or even nationally accepted normal reference range and cut-off value of the intrapancreatic lipid content and pancreatic steatosis, respectively. There is a remarkable variability (3.6–10%) in reported pancreatic lipid contents of control groups in different studies [5, 8–17]. Therefore, there are neither diagnostic recommendations, nor treatment guidelines of pancreatic steatosis despite that there is accumulating evidence that it might have a significant role in the development of T2DM.

A few studies raised that the association between liver and pancreatic fat content could be confounded by major factors [9, 10] therefore we aimed to assess this association stratified by *PNPLA3* rs738409 (missense variant), a major genetic risk factor for NAFLD development.

Pancreatic lipid accumulation is associated with beta-cell dysfunction [12, 16], particularly in patients with increased susceptibility to impaired beta-cell function due to the presence of T2DM risk gene variants [18]. It is likely that the islet pathology and subsequent metabolic consequences also depend on the infiltrating (pre)adipocyte induced pro-inflammatory changes [19].

To the best of our knowledge there is no available public report on the effect of individual T2DM risk gene variants in combination with pancreatic fat on β-cell function. We selected *TCF7L2* rs7903146 (intron variant) as the strongest T2DM associated genetic risk factor to date that is also associated with higher fasting and post-challenge glucose levels in Caucasians and another β-cell dysfunction associated T2DM risk variant (*WFS1* rs1801214, missense) and also *11ΒHSD1* rs4844880 as a reported visceral obesity associated intron variant for further analysis from a prior gestational diabetes mellitus (GDM)-genetic study [20–29].

## 2. Methods

### 2.1 Study population

This study was focused on the pancreatic lipid accumulation and was conducted complementary to a recently completed post-GDM genetic based recall study on metabolic (dysfunction) associated fatty liver disease (MAFLD) [30]. Thirty-nine Hungarian women prone to T2DM were enrolled based on the *PNPLA3* rs738409 genotype (only homozygotes included) to the study focused primarily on the liver lipid accumulation and all of them underwent a detailed metabolic phenotyping between 2016 and 2020.

None of the participants were previously diagnosed with type 2 diabetes mellitus or treated with a glucose or a lipid lowering medication at the enrolment.

### 2.2 Exclusion criteria

MR incompatible metal implants and a maximum body weight of 200 kg, waist/hip circumference < 135 cm/148 cm, drug treatment with known impact on the glycaemic traits, significant alcohol consumption (> 20 g/day), and presence of a malignant disease were considered as exclusion criteria. In addition, patients with other major chronic/acute diseases, ongoing pregnancy/breastfeeding or with contraindication for OGTT were also excluded.

### 2.3 Genotyping

The genotypes of the gene variants: *PNPLA3* rs738409; *TCF7L2* rs7903146; *WFS1* rs1801214; *11ΒHSD1* rs4844880 were extracted for this analysis from larger genotype database established during a preceding study [29]. These gene variants were selected based on their previously reported associations with relevant traits rs738409: NAFLD development; rs7903146, rs1801214: T2DM development, β-cell dysfunction; and rs4844880: visceral obesity. All the gene variants assessed occurred with a minor allele frequency (MAF) of at least 0.20 in the Hungarian population [29].

### 2.4 Clinical data

Anthropometric data (weight[kg], height[m], waist/hip circumferences[cm], BMI, waist/hip ratio [W/H]), age, medical history (including GDM) were recorded. Based on the result of 75 g OGTT and/or HemoglobinA_1c_ (HbA1c) the diagnosis of diabetes mellitus (DM) or prediabetes (IFG/IGT) was established according to the WHO, 2016 ADA and the Hungarian guidelines [31–33]. MAFLD was diagnosed based on the recently proposed criteria [30, 34].

### 2.5 75 g OGTT, metabolic phenotyping and measurement of β-cell function

Seventy-five gramm OGTT (0′-30′-120′) was performed after an overnight fasting period of at least 10 h. Plasma glucose (PG), HbA1c, and other routine laboratory parameters were determined according to standard protocols and the International Federation of Clinical Chemistry (IFCC) standards. Serum insulin levels were measured using Liaison analyser (DiaSorin, Saluggia, Italy). HOMA2-IR, Triglyceride-Glucose index (TyG) as surrogate measures of insulin resistance and HOMA2-B values as marker of β-cell function were calculated [35]. TyG index was calculated as: TyG = ln[ 38,67*Tg(mg/dl)*18*glucose(mg/dl)/2] [36–38]. In order to assess the future T2DM development risk we used the following markers: Disposition Index basal (DI_basal_: HOMA2-B x 1/HOMA2-IR) [39, 40], Insulin Secretion and Sensitivity Index-2 (ISSI-2: using the formula: AUC_Insulin_/AUC_Glucose_ x Matsuda) [41].

### 2.6 Determination of intrapancreatic and intrahepatic lipid content

Hepatic and pancreatic triglyceride contents (HTGC and PTGC, respectively) were determined as follows: All MR imaging (MRI) sessions were acquired on a clinical 3T MRI system (Prisma, Siemens Healthineers, Erlangen, Germany) with the subject in a supine position. For MRI protocol, standard body-array 18 channel flexible coils were positioned on the liver and pancreatic region and combined with the spine array coil located below the subject. Imaging proton density fat-fraction (PDFF), unenhanced axial images were obtained by using a low–flip-angle, six-echo two-dimensional spoiled gradient-recalled-echo sequence with all array coil elements (TE = 2,4.1,6.2,8.2,10.2 and 12.3 ms). The repetition time and flip angle were chosen to avoid T1 weighting: TR: 15 ms; Flip-angle: 11°;FOV = 240 × 400 mm in plane; matrix = 240 × 130; slice thickness = 3.5 mm, space between slices 4.3 mm. Phase and magnitude images were systematically saved.

MR imaging: Multi-section PDFF maps were generated offline from the source images via joint estimation of water and fat images and field maps using custom made MATLAB routines [29]. The method used complex signal model similar to advanced multipoint DIXON/IDEAL algorithms (incorporating multipeak/multifrequency fat model and T2* decay) analysing six-echo FLASH complex images. Voxels representing pancreas tissue were delineated with a freehand ROI defining tool by an expert radiologist on multi-section PDFF maps excluding ill-defined edges (due to macroscopic fatty infiltration e.g.), vessels, the pancreatic duct, and artifacts. In each subject the mean value of the selected voxels was calculated and this value represented the average pancreatic fat fraction.

### 2.7 Statistical analysis

Data distributions and comparisons between two or more cohorts were assessed using the Shapiro-Wilk’s, Mann-Whitney-U and Kruskal-Wallis tests, respectively. Spearman’s rank-correlation was also used. Linear regression model was employed for interaction analysis. Genotype based subgroups of T2DM associated genes were assessed using the dominant model for the minor allele. Case data were deleted in the given analysis when data were missing. Benjamini-Hochberg p-value correction was used to control false discovery rate in multiple testing. “R” (version 4.0.4, R Foundation for Statistical Computing, Vienna, Austria, 2021) and TIBCO Statistica (version 13.4.0.14, TIBCO Software Inc.) softwares were used.

Genotype distributions were non-deviant from the Hardy-Weinberg equilibrium (HWE) in the original study [29], and HWE was not assessed in this analysis.

## 3. Results

### 3.1 Study population characteristic

Clinical characteristics of the study population are presented in Table 1. Continuous data are presented as medians and 25–75th percentiles due to that most of the variables were non-normally distributed and due to the low sample size. None of the participants had pancreatitis in their personal medical history. All patients were naïve for antidiabetic and lipid lowering drug therapy.

**Table 1:** Population characteristic

	Median (25–75th percentile) or Number of patients (%)
Age (years)	37.0 (34.0–40.0)
BMI (kg/m^2^)	26.2 (22.8–32.4)
Overweight + obesity	24 (61.5%)
GDM history	22 (56.4%)
HbA1c % [mmol/mol]	5.5[37] (5.2[33]-5.6[38])
Prediabetes^a^ + T2DM	14 (35.9%)
PTGC (%)	8.2 (5.4–10.2)
HTGC (%)	3.9 (2.8–8.4)
MAFLD	14 (35.9%)

*GDM* gestational diabetes mellitus, *T2DM *type 2 diabetes mellitus, *PTGC* pancreatic triacylglycerol content, *HTGC* hepatic triacylglycerol content, *MAFLD* metabolic (dysfunction) associated fatty liver disease

^a^Diagnosed by using HbA1c and/or glucose values from OGTT (IFG, IGT)

### 3.2 PTGC vs. BMI, age

A significant correlation was observed between PTGC and BMI (R = 0.40 p = 0.012), in contrast to that of between PTGC and age (R = 0.12 p = 0.440). Waist circumference also correlated with PTGC (R = 0.50 p = 0.002).

### 3.3 PTGC vs. HTGC and its stratification by *PNPLA3* rs738409 genotype

**Fig. 1:**
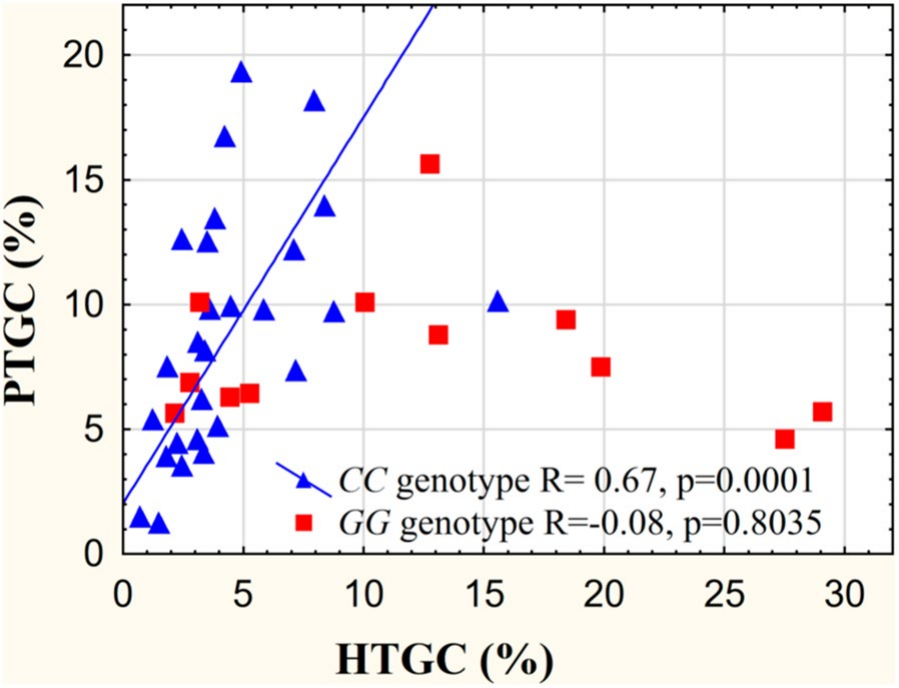
Correlation between PTGC and HTGC values stratified by *PNPLA3* rs738409 genotype. Abbreviations: PTGC: pancreatic triacylglycerol content, HTGC: hepatic triacylglycerol content

We found a significant correlation between HTGC and PTGC values in the entire study population (R = 0.46 p = 0.004), and in the *PNPLA3* rs738409 *CC* genotype group after stratification by the genotype (R = 0.67 p = 10^− 4^) but not in the *GG* group (R=-0.08 p = 0.80) (Fig. 1).

### 3.4 IPCL vs. HbA1c, OGTT derived markers of insulin resistance and disposition indices

**Fig. 2:**
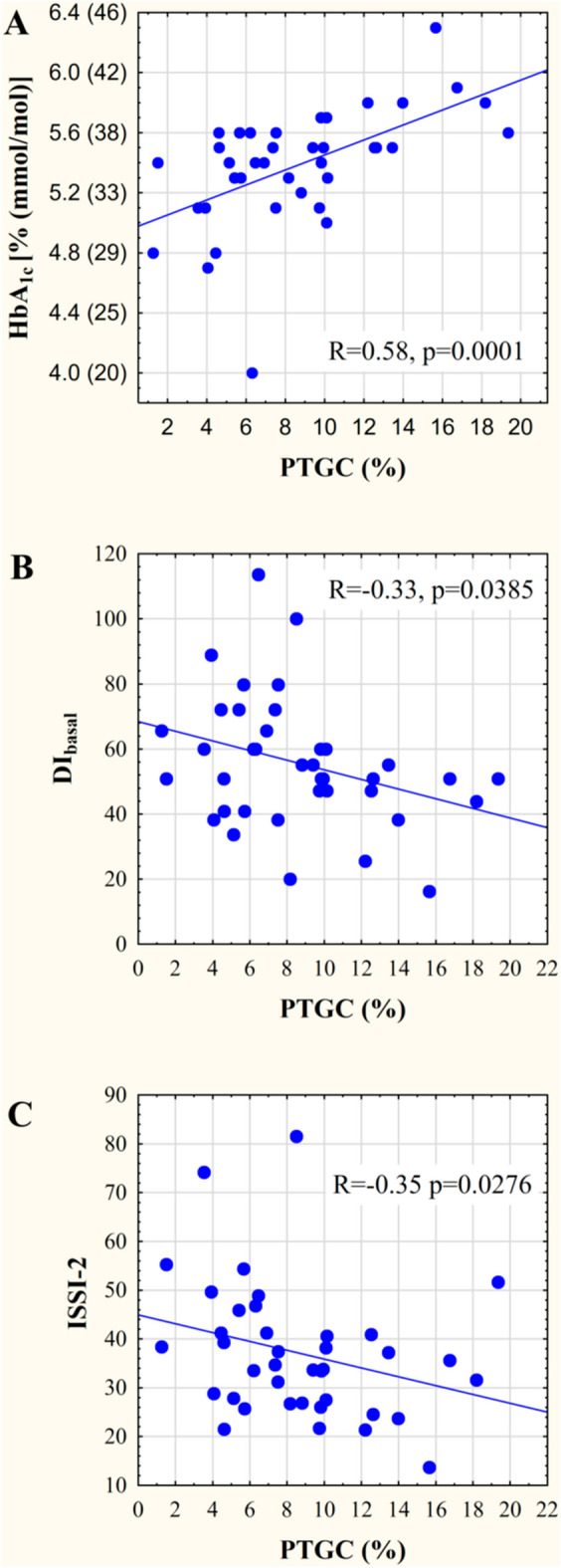
Correlations among PTGC and HbA1c and disposition indices. **A** Between PTGC and HbA1c values, **B** PTGC and DI_basal_, **C** PTGC and ISSI-2. Abbreviations: PTGC: pancreatic triacylglycerol content, DI_basal_: disposition index basal, ISSI-2: insulin secretion sensitivity index − 2

PTGC and HbA1c values were highly correlated (R = 0.58 p = 10^− 4^, Fig. 2A). No significant correlations were found between HbA1c and HTGC levels, BMI or waist circumference.

Negative correlations were observed between PTGC and disposition indices: DI basal value (R= − 0.33, p = 0.039) derived from fasting steady state insulin and glucose values (Fig. 2B) and ISSI-2 value (R= −0.35, p = 0.028) derived from OGTT (Fig. 2C).

**Fig. 3:**
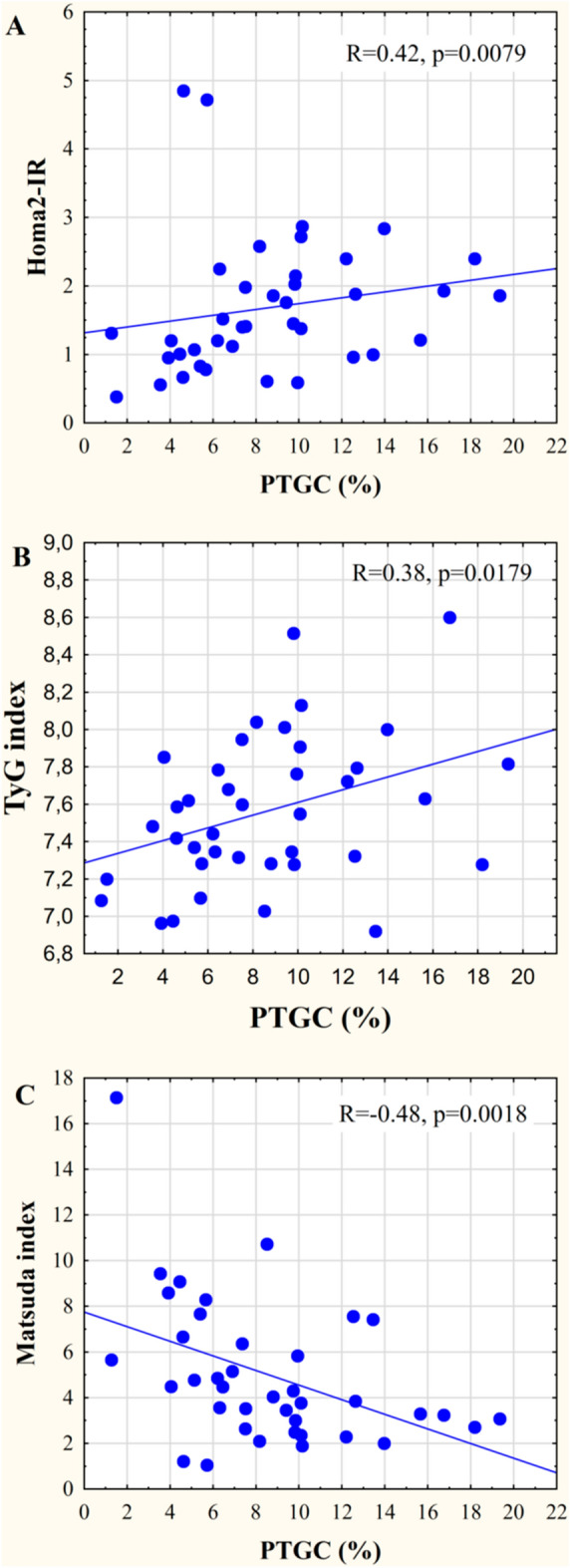
Correlations between PTGC and surrogate markers of insulin resistance/sensitivity. **A** PTGC and HOMA-2IR, **B** PTGC and TyG index, **C** PTGC and Matsuda index. Abbreviations: PTGC: pancreatic triacylglycerol content, TyG index: triglyceride-glucose index

PTGC values were also correlated with surrogate markers of insulin resistance (HOMA2-IR: R = 0.42, p = 0.008, TyG: R = 0.38, p = 0.018, Fig. 3 A, B, respectively), and inversely with the “whole body” sensitivity index (Matsuda index: R= − 0.48, p = 0.002; indicated on Fig. 3C).

In addition, there was a correlation between PTGC and serum triglyceride levels with borderline significance (R = 0.34, p = 0.035). In contrast no correlations were found between PTGC and total cholesterol levels or its fractions (HDL, LDL).

### 3.5 Effect of intrapancreatic lipid content in combination with risk gene variants on surrogate markers of insulin secretion

**Fig. 4:**
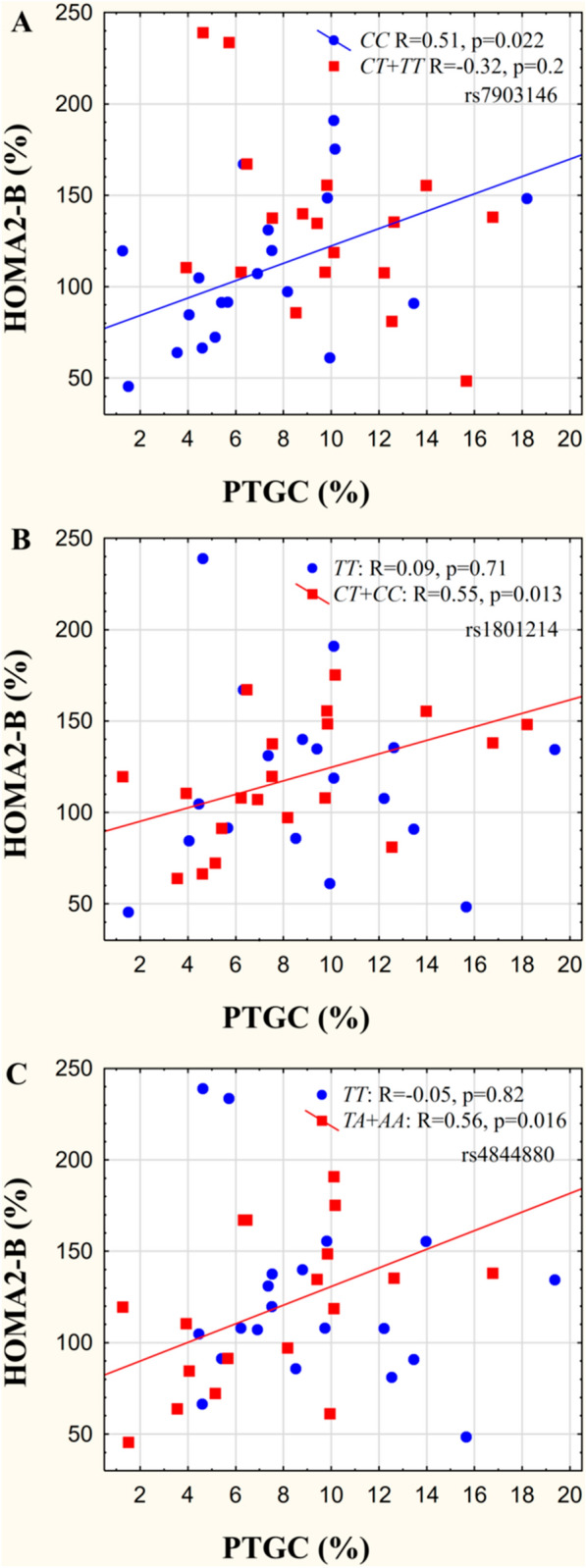
Correlation between PTGC and insulin secretion marker HOMA2-B stratified by genotypes of T2DM risk gene variants. **A** *TCF7L2* rs12243326, **B** *WFS-1* rs1801214, **C** *11ΒHSD1* rs4844880. Abbreviations: PTGC: pancreatic triacylglycerol content

Although we could not detect any correlation between the pancreatic fat content and surrogate markers of β-cell function (HOMA2-B, AUC_insulin_/AUC_glucose_) in the entire study population a specific pattern of correlations was identified when the results were stratified by the genotypes (Table 2; Fig. 4 A, B, and  C). Subsequently we performed an interaction analysis between pancreatic lipid values and T2DM / visceral obesity risk genotypes on β-cell function indices. We found significant interactions between the PTGC and variants of *TCF7L2* and *11ΒHSD1* (Table 2). Higher PTGC values were found in patients carrying the risk (*T*) allele of the *TCF7L2* variant (rs7903146 dominant model p = 0.050), and subjects with the “*TT*” genotype tended to have the highest values (additive model: Kruskal-Wallis p = 0.089) without any difference in BMI values.

**Table 2:** Correlations between PTGC and indices of -cell function stratified by type T2DM associated gene variants

Indices of β-cell function	Gene	Variant ID	Genotype^a^ (n)	Correlation			Interaction analysis^b^
				R	p_(crude/corrected_^c^_)_	Power	p_(crude/ corrected_^c^_)_
HOMA2-B	*TCF7L2*	rs7903146	*CC* (20)	0.51	0.022/0.044	0.664	0.001/0.003
			*CT* + *TT* (18)	− 0.32	0.20/0.20	0.263	
	*WFS1*	rs1801214	*TT*^d^ (18)	0.09	0.71/0.71	0.065	0.18/ 0.18
			*CT*^d^ + *CC* (20)	0.55	0.013/0.044	0.745	
	*11ΒHSD1*	rs4844880	*TT* (20)	− 0.05	0.82/0.82	0.055	0.029/0.044
			*TA* + *AA* (18)	0.56	0.016/0.044	0.714	
AUC_insulin_/AUC_glucose_	*TCF7L2*	rs7903146	*CC* (20)	0.39	0.087/0.17	0.415	0.013/0.039
			*CT* + *TT* (18)	− 0.21	0.40/0.60	0.136	
	*WFS1*	rs1801214	*TT*^*d*^(18)	0.13	0.61/0.73	0.082	0.81/0.81
			*CT*^*d*^ + *CC* (20)	0.51	0.021/0.13	0.664	
	*11ΒHSD1*	rs4844880	*TT* (20)	− 0.04	0.87/0.87	0.053	0.27/0.41
			*TA* + *AA* (18)	0.47	0.048/0.14	0.530	

*PTGC* Pancreatic Triacylglycerol Content, *T2DM* type 2 diabetes mellitus, *HOMA2-B* Homeostasis Model Assessment 2-B, *AUC* Area Under the Curve

^a^Major allele homozygous vs. minor allele carriers;

^b^Linear regression model. Outcome: insulin secretion marker, genotype in a dominant model (for minor allele) as a categorical and PTGC as a continuous variable. Interaction: categorical x continuous variable;

^c^Benjamini-Hochberg (FDR) p-correction;

^d^Only *C* and *T* alleles detected in the Hungarian-Austrian cohort; multiallelic in GRCh38 assembly using the Ensembl browser “1000 Genomes” database [55]

In case of the *TCF7L2* gene variant we observed a statistically significant correlation and interaction indicating that the β-cell function is increased in parallel with the increasing PTGC in patients without the risk allele indicating a preserved β-cell compensatory mechanism for the increased insulin resistance. The other studied gene variants (*WFS-1*, *11ΒHSD1*) also displayed correlation patterns referring to a loss of β-cell compensatory mechanism in patients homozygous for the risk alleles.

## 4. Discussion

In this study we recalled middle aged female participants from a prior GDM genetic association study [29]. All participants underwent detailed clinical phenotyping and MRI assessment of liver and whole pancreatic proton density fat fractions. A significant correlation was found between HTGC and PTGC values in the entire study population, notably in participants with the *PNPLA3* rs738409 *CC* genotype. We first report a strong correlation between the pancreatic lipid content and the HbA1c values in antidiabetic drug naïve individuals who are prone to type 2 diabetes mellitus development. We also observed significant correlations between PTGC and BMI, waist circumference, surrogate markers of insulin resistance (HOMA2-IR, TyG) and indices indicative for future T2DM development (DI_basal_, ISSI-2). Furthermore, we found correlations between PTGC and HOMA2-B (marker of insulin secretion) provided that the results were stratified by known T2DM risk gene variants associated to β-cell dysfunction (*TCF7L2*, *WFS1*) and a gene variant associated to visceral obesity (*11ΒHSD1*) .

Obesity is a common risk factor for both fatty liver disease and pancreatic fat accumulation [4], however the literature was not uniformly consistent about the presence of a direct relationship between pancreatic and liver fat accumulations [8–10, 15, 16]. In addition to confirming the correlations between PTGC and anthropometric data (BMI, waist circumference) here we first report a significant positive correlation between PTGC and HTGC values only in participants with the *PNPLA3* rs738409 *CC* genotype. According to our result, this parallel fat accumulation in the two organs occured only when the liver fat values were not shifted by the effect of *PNPLA3* risk variant, indicating that the liver and pancreatic fat accumulations were likely related to a common metabolic origin. Our finding contributes to the understanding of the divergent prior reports about the only moderate correlation or the lack of correlation between PTGC and HTGC values without stratification by the *PNPLA3* risk genotype [8–10, 15, 16].

A prior study already reported a step-wise consecutive increase in PTGC in subjects with normal glucose tolerance (NGT) vs. prediabetes vs. T2DM in an European population with over 350 participants [17]. Smaller studies reported similar findings [9, 16, 42]. Furthermore, bariatric surgery studies indicated that the loss of pancreatic fat is significantly associated with the reversal of T2DM [5, 8]. More specifically, the presurgery PTGC was increased in patients with T2DM and the PTGC reduced to similar levels that was observed in the NGT group and this was associated with the improvement in the first phase insulin response 8 weeks after surgery [5].There were inconsistent reports on the associations between PTGC and HbA1c, due to that two prior studies [8, 9] reported associations, but another study [42] with higher sample size did not observe any correlation. The inconsistency in prior reports may be related to the potential glucose and lipid lowering drug use, in contrast to our study where only drug-naïve participants were enrolled. This study design enabled us to assess the natural disease course of type 2 diabetes mellitus (NGT-prediabetes-T2DM) in early stages in a diabetes prone population due to the enriched presence of participants with prior GDM. The clinical relevance is that HbA1c is a well-known, stable blood based marker used in diabetes care for monitoring and also to establish the diagnosis of both prediabetes and diabetes [43]. Disposition indices (DI_basal_ and ISSI-2) that are known predictors of future T2DM development [44–46] were also correlated with PTGC. Although all three correlations were statistically significant, the correlation between PTGC and HbA1c was stronger compared to that of with the disposition indices. This may be related to that HbA1c is indicative for the mean glucose value in the prior 2–3 months, in contrast to disposition indices that are calculated from the glucose and insulin levels characterized with a lower reproducibility during OGTT [46, 47].

The current understanding of T2DM development is that increased insulin resistance is a key pathologic step in early stages when the β-cell dysfunction is not yet preponderant and the insulin overproduction can still cope with the higher demand. Subsequently the β-cell dysfunction turns into the major drive of the disease progression and as a consequence the glycaemic control is lost [48].

The observed associations between the higher BMI, HOMA2-IR, TyG values and higher pancreatic lipid content indicated a higher insulin need and the related compensatory increase in β-cell function in these individuals.

A recent multi-organ and multi-trait GWAS reported associations between a few gene variants and pancreatic fat content derived from abdominal MRI scans [49]. We could assess the effect of other gene variants (*TCF7L2* rs7903146, *WFS1* rs1801214 and *11ΒHSD1* rs4844880) that were available from our prior study [29] and including core gene variants associated with T2DM, β-cell dysfunction and visceral adiposity [20–26, 28].

Although the general concept of the combined effect of T2DM genetic risk factors and pancreatic lipid content on insulin secretion was successfully proposed [18] to our best knowledge no direct interaction between *TCF7L2* rs7903146 risk variant and pancreatic fat content was described earlier. Our results first indicate that the β-cell function (HOMA2-B) increase observed in the *TCF7L2* rs7903146 “*CC*” homozygotes as part of an adequate adaptation process becomes disrupted in “*T*” risk allele carriers and this difference develops to be higher along with the increase of PTGC early during the course of T2DM development.

Pancreatic fat accumulation could be an auxiliary factor for the genetic susceptibility linked to the rs7903146 variant which until date shows the strongest association with T2DM across different populations [20]. The *TCF7L2* gene encodes a transcription factor in the WNT signalling pathway and it is involved in vital functions in the pancreatic islets: pancreas development, β cell mass maintenance, secretory functions of matured β-cells, regulation of insulin production and processing [21, 22]. It was reported that the rs7903146 “*T”* (risk) allele carriers had an elevated *TCF7L2* mRNA expression in the human pancreatic islets [21].

One explanation could be that the excess pancreatic lipid deposition is occuring in parallel with the intra-islet fat accumulation and this may alter the islet cells’ microenvironment by increasing the exposition of β-cells to free fatty acids and proinflammatory cytokines resulting in a direct pathological effect. The combined effect of *WFS1* gene variant and PTGC on HOMA2-B may potentially be explained by a similar pathology on β cell survival [26].

This mechanism might be particularly relevant when the β-cells are prone to dysfunction due to the genetic susceptibility [50]. The effect of the *11ΒHSD1* gene variant on β-cells is less well characterized, however it is one of the “disallowed” genes in β-cells and the variant’s effect on the function and maintenance cannot be excluded [51].

Based on the result with borderline statistical significance, it may also be raised that the rs7903146, the strongest confirmed T2DM risk gene variant [20, 52] might have a direct effect on the pancreatic lipid content, however, our results are not yet fully conclusive, and should be replicated in studies with larger sample sizes. Nevertheless, it was already reported that the *TCF7L2* rs7903146 risk (“*T*”) allele had a negative effect on BMI, total body fat and visceral fat reductions after lifestyle intervention in a human trial and this result is consistent with baseline observations made in a heterozygous *TCF7L2* knock out animal model (*Tcf7l2*^+/−^) [53, 54].

The limitations of our findings include the post-hoc nature of our study, the limited sample size and also that the diet, physical activity, family history, and hormonal fluctuations were not considered.

## Data Availability

The datasets used and analysed during the current study are available from the corresponding author on reasonable request.

## Abbreviations

T2DM: Type 2 diabetes mellitus
NAFLD: Non-alcoholic fatty liver disease
MR: Magnetic resonance
GDM: Gestational diabetes mellitus
BMI: Body Mass Index
MAF: Minor allele frequency
W/H: Waist to Hip ratio
OGTT: Oral glucose tolerance test
HbA1c: HemoglobinA1c
DM: Diabetes mellitus
IFG: Impaired fasting glucose
IGT: Impaired glucose tolerance
WHO: World Health Organisation
ADA: American Diabetes Association
MAFLD: Metabolic (dysfunction) associated fatty liver disease
PG: Plasma glucose
IFCC: International Federation of Clinical Chemistry
HOMA: Homeostasis Model Assessment
TyG: Trygliceride-Glucose Index
DI: Disposition Index
ISSI-2: Insulin Secretion and Sensitivity Index − 2
AUC: Area Under the Curve
HTGC: Hepatic triacylglycerol content
PTGC: Pancreatic triacylglycerol content
MRI: MR imaging
PDFF: Proton density fat fraction
HWE: Hardy-Weinberg equilibrium
NGT: Normal glucose tolerance

## References

[1] Ogilvie RF (1933). The islands of langerhans in 19 cases of obesity. J Pathol Bacteriol.

[2] Younossi ZM, Koenig AB, Abdelatif D, Fazel Y, Henry L, Wymer M (2016). Global epidemiology of nonalcoholic fatty liver disease-meta-analytic assessment of prevalence, incidence, and outcomes. Hepatology.

[3] Catanzaro R, Cuffari B, Italia A, Marotta F (2016). Exploring the metabolic syndrome: nonalcoholic fatty pancreas disease. World J Gastroenterol.

[4] Wagner R, Eckstein SS, Yamazaki H, Gerst F, Machann J, Jaghutriz BA (2022). Metabolic implications of pancreatic fat accumulation. Nat Rev Endocrinol.

[5] Steven S, Hollingsworth KG, Small PK, Woodcock SA, Pucci A, Aribisala B (2016). Weight loss decreases excess pancreatic triacylglycerol specifically in type 2 diabetes. Diabetes Care.

[6] Firneisz G (2014). Non-alcoholic fatty liver disease and type 2 diabetes mellitus: the liver disease of our age?. World J Gastroenterol.

[7] European Association for the Study of the Liver (EASL)European Association for the Study of Diabetes (EASD)European Association for the Study of Obesity (EASO) (2016). EASL–EASD–EASO clinical practice guidelines for the management of non-alcoholic fatty liver disease. J Hepatol.

[8] Gaborit B, Abdesselam I, Kober F, Jacquier A, Ronsin O, Emungania O (2015). Ectopic fat storage in the pancreas using ^1^H-MRS: importance of diabetic status and modulation with bariatric surgery-induced weight loss. Int J Obes.

[9] Kato S, Iwasaki A, Kurita Y, Arimoto J, Yamamoto T, Hasegawa S (2019). Three-dimensional analysis of pancreatic fat by fat-water magnetic resonance imaging provides detailed characterization of pancreatic steatosis with improved reproducibility. PLoS ONE.

[10] Hannukainen JC, Borra R, Linderborg K, Kallio H, Kiss J, Lepomäki V (2011). Liver and pancreatic fat content and metabolism in healthy monozygotic twins with discordant physical activity. J Hepatol.

[11] Kühn J-P, Berthold F, Mayerle J, Völzke H, Reeder SB, Rathmann W (2015). Pancreatic steatosis demonstrated at MR imaging in the general population: clinical relevance. Radiology.

[12] Heni M, Machann J, Staiger H, Schwenzer NF, Peter A, Schick F (2010). Pancreatic fat is negatively associated with insulin secretion in individuals with impaired fasting glucose and/or impaired glucose tolerance: a nuclear magnetic resonance study. Diabetes Metab Res Rev.

[13] van der Zijl NJ, Goossens GH, Moors CCM, van Raalte DH, Muskiet MHA, Pouwels PJW (2011). Ectopic fat storage in the pancreas, liver, and abdominal fat depots: impact on β-cell function in individuals with impaired glucose metabolism. J Clin Endocrinol Metab.

[14] Koyuncu Sokmen B, Sahin T, Oral A, Kocak E, Inan N (2021). The comparison of pancreatic and hepatic steatosis in healthy liver donor candidates. Sci Rep.

[15] Patel NS, Peterson MR, Lin GY, Feldstein A, Schnabl B, Bettencourt R (2013). Insulin resistance increases MRI-estimated pancreatic fat in nonalcoholic fatty liver disease and normal controls. Gastroenterol Res Pract.

[16] Tushuizen ME, Bunck MC, Pouwels PJ, Bontemps S, van Waesberghe JHT, Schindhelm RK (2007). Pancreatic fat content and β-cell function in men with and without type 2 diabetes. Diabetes Care.

[17] Heber SD, Hetterich H, Lorbeer R, Bayerl C, Machann J, Auweter S (2017). Pancreatic fat content by magnetic resonance imaging in subjects with prediabetes, diabetes, and controls from a general population without cardiovascular disease. PLoS ONE.

[18] Wagner R, Jaghutriz BA, Gerst F, Barroso Oquendo M, Machann J, Schick F (2020). Pancreatic steatosis associates with impaired insulin secretion in genetically predisposed individuals. J Clin Endocrinol Metab.

[19] Gerst F, Wagner R, Kaiser G, Panse M, Heni M, Machann J (2017). Metabolic crosstalk between fatty pancreas and fatty liver: effects on local inflammation and insulin secretion. Diabetologia.

[20] Prasad RB, Groop L (2015). Genetics of type 2 diabetes—pitfalls and possibilities. Genes.

[21] Lyssenko V, Lupi R, Marchetti P, Guerra SD, Orho-Melander M, Almgren P (2007). Mechanisms by which common variants in the *TCF7L2* gene increase risk of type 2 diabetes. J Clin Invest.

[22] Zhou Y, Park S-Y, Su J, Bailey K, Ottosson-Laakso E, Shcherbina L (2014). TCF7L2 is a master regulator of insulin production and processing. Hum Mol Genet.

[23] Cropano C, Santoro N, Groop L, Dalla Man C, Cobelli C, Galderisi A (2017). The rs7903146 variant in the *TCF7L2* gene increases the risk of prediabetes/type 2 diabetes in obese adolescents by impairing β-cell function and hepatic insulin sensitivity. Diabetes Care.

[24] Kondo M, Tanabe K, Amo-Shiinoki K, Hatanaka M, Morii T, Takahashi H (2018). Activation of GLP-1 receptor signalling alleviates cellular stresses and improves beta cell function in a mouse model of Wolfram syndrome. Diabetologia.

[25] Schäfer SA, Müssig K, Staiger H, Machicao F, Stefan N, Gallwitz B (2009). A common genetic variant in WFS1 determines impaired glucagon-like peptide-1-induced insulin secretion. Diabetologia.

[26] Mahajan A, Taliun D, Thurner M, Robertson NR, Torres JM, Rayner NW (2018). Fine-mapping type 2 diabetes loci to single-variant resolution using high-density imputation and islet-specific epigenome maps. Nat Genet.

[27] Molnár Á, Kövesdi A, Szücs N, Tóth M, Igaz P, Rácz K (2016). Polymorphisms of the GR and HSD11B1 genes influence body mass index and weight gain during hormone replacement treatment in patients with Addison’s disease. Clin Endocrinol.

[28] Lutz SZ, Peter A, Machicao F, Lamprinou A, Machann J, Schick F (2016). Genetic variation in the 11β-hydroxysteroid-dehydrogenase 1 gene determines NAFLD and visceral obesity. J Clin Endocrinol Metab.

[29] Rosta K, Al-Aissa Z, Hadarits O, Harreiter J, Nádasdi Á, Kelemen F (2017). Association study with 77 SNPs confirms the robust role for the rs10830963/G of MTNR1B variant and identifies two novel associations in gestational diabetes mellitus development. PLoS ONE.

[30] Nádasdi Á, Gál V, Harreiter J, Rosta K, Kautzky-Willer A, Igaz P (2020). Effect of PNPLA3 rs738409 genotype and gestational diabetes history on fasting glucagon levels in early NAFLD. Diabetologia.

[31] World Health Organization. World Health Organization: definition and diagnosis of diabetes mellitus and intermediate hyperglycaemia. Report of a WHO consultation. 2006. https://www.who.int/publications/i/item/definition-and-diagnosis-of-diabetes-mellitus-and-intermediate-hyperglycaemia. Accessed 24 July 2022.

[32] American Diabetes Association (2016). Classification and diagnosis of diabetes. Section 2. In standards of medical care in diabetes. Diabetes Care.

[33] Gaál Z, Gerő L, Hidvégi T, Jermendy G, Kempler P, Winkler G (2017). Clinical practice guideline – diagnosis, antihyperglycaemic treatment and care of patients with diabetes in adulthood. Diabetol Hung.

[34] Eslam M, Newsome PN, Sarin SK, Anstee QM, Targher G, Romero-Gomez M (2020). A new definition for metabolic dysfunction-associated fatty liver disease: an international expert consensus statement. J Hepatol.

[35] Levy JC, Matthews DR, Hermans MP (1998). Correct Homeostasis Model Assessment (HOMA) evaluation uses the computer program. Diabetes Care.

[36] Lee J, Kim B, Kim W, Ahn C, Choi HY, Kim JG (2021). Lipid indices as simple and clinically useful surrogate markers for insulin resistance in the U.S. population. Sci Rep.

[37] Guerrero-Romero F, Villalobos-Molina R, Jiménez-Flores JR, Simental-Mendia LE, Méndez-Cruz R, Murguía-Romero M (2016). Fasting triglycerides and glucose index as a diagnostic test for insulin resistance in young adults. Arch Med Res.

[38] Alizargar J, Hsieh N-C, Wu S-FV (2020). The correct formula to calculate triglyceride-glucose index (TyG). J Pediatr Endocrinol Metab.

[39] Cobelli C, Toffolo GM, Man CD, Campioni M, Denti P, Caumo A (2007). Assessment of β-cell function in humans, simultaneously with insulin sensitivity and hepatic extraction, from intravenous and oral glucose tests. Am J Physiol Endocrinol Metab.

[40] Caumo A, Perseghin G, Brunani A, Luzi L (2006). New insights on the simultaneous assessment of insulin sensitivity and β-cell function with the HOMA2 method. Diabetes Care.

[41] Retnakaran R, Shen S, Hanley AJ, Vuksan V, Hamilton JK, Zinman B (2008). Hyperbolic relationship between insulin secretion and sensitivity on oral glucose tolerance test. Obesity.

[42] Heiskanen MA, Motiani KK, Mari A, Saunavaara V, Eskelinen J-J, Virtanen KA (2018). Exercise training decreases pancreatic fat content and improves beta cell function regardless of baseline glucose tolerance: a randomised controlled trial. Diabetologia.

[43] American Diabetes Association (2021). 2. Classification and Diagnosis of diabetes: standards of medical care in diabetes*—*2021. Diabetes Care.

[44] Matsuda M (2010). Measuring and estimating insulin resistance in clinical and research settings. Nutr Metab Cardiovasc Dis.

[45] Lorenzo C, Wagenknecht LE, Rewers MJ, Karter AJ, Bergman RN, Hanley AJG (2010). Disposition index, glucose effectiveness, and conversion to type 2 diabetes. Diabetes Care.

[46] Kramer CK, Vuksan V, Choi H, Zinman B, Retnakaran R (2014). Emerging parameters of the insulin and glucose response on the oral glucose tolerance test: Reproducibility and implications for glucose homeostasis in individuals with and without diabetes. Diabetes Res Clin Pract.

[47] Ko GT, Chan JC, Woo J, Lau E, Yeung VT, Chow CC (1998). The reproducibility and usefulness of the oral glucose tolerance test in screening for diabetes and other cardiovascular risk factors. Ann Clin Biochem.

[48] Tabák A, Jokela M, Akbaraly T, Brunner E, Kivimäki M, Witte D (2009). Trajectories of glycemia, insulin sensitivity and insulin secretion preceding the diagnosis of type 2 diabetes: the Whitehall II study. Lancet.

[49] Liu Y, Basty N, Whitcher B, Bell JD, Sorokin EP, van Bruggen N (2021). Genetic architecture of 11 organ traits derived from abdominal MRI using deep learning. eLife.

[50] Charles MA, Leslie RD, Diabetes (2021). Concepts of β-cell organ dysfunction and failure would lead to earlier diagnoses and prevention. Diabetes.

[51] Pullen TJ, Huising MO, Rutter GA (2017). Analysis of purified pancreatic islet beta and alpha cell transcriptomes reveals 11β-hydroxysteroid dehydrogenase (Hsd11b1) as a novel disallowed gene. Front Genetics.

[52] Fuchsberger C, Flannick J, Teslovich TM, Mahajan A, Agarwala V, Gaulton KJ (2016). The genetic architecture of type 2 diabetes. Nature.

[53] Haupt A, Thamer C, Heni M, Ketterer C, Machann J, Schick F (2010). Gene variants of TCF7L2 influence weight loss and body composition during lifestyle intervention in a population at risk for type 2 diabetes. Diabetes.

[54] Yang H, Li Q, Lee J-H, Shu Y (2012). Reduction in *Tcf7l2* expression decreases diabetic susceptibility in mice. Int J Biol Sci.

[55] Howe KL, Achuthan P, Allen J, Allen J, Alvarez-Jarreta J, Amode MR (2021). Ensembl 2021. Nucleic Acids Res.

